# The Role of N-acetylcysteine in the Treatment of Accidental Submersion of the Hands in Liquid Nitrogen

**DOI:** 10.7759/cureus.18129

**Published:** 2021-09-20

**Authors:** Erin Orozco, Alysa Birnbrich, Shari R Liberman

**Affiliations:** 1 Orthopedic Surgery, University of Texas Health Science Center at Houston, Houston, USA; 2 Orthopedic Surgery, Houston Methodist Hospital, Houston, USA; 3 Orthopedics and Sports Medicine, Houston Methodist Hospital, Houston, USA

**Keywords:** burn injury, liquid nitrogen, hand injuries, n-acetylcysteine, hand surgery

## Abstract

N-acetylcysteine (NAC) is a compound with numerous uses, especially in cases which require prevention of cellular damage. To the authors’ knowledge, no reports of NAC as treatment for liquid nitrogen (LN2) injuries currently exist. We present a case in which a 40-year-old woman accidentally submerged her hands in LN2 while working in a lab. The patient was treated with NAC, antibiotics, and wound care. Six months post-injury, the patient had full range of motion, full sensation, full function, and no pain. Therefore, NAC, in combination with dressing changes and antibiotics, can be used to successfully treat patients with LN2 burns.

## Introduction

Liquid nitrogen (LN2) is a colorless, low viscosity liquid that is a compact and easily transported source of dry nitrogen gas [1]. It has a wide range of applications, such as serving as a coolant for computers, removing cancerous skin cells, or instant freezing of foods or drinks [1]. However, due to the extreme low temperature of the liquid, careless handling of the material or any object cooled by it can result in thermal burns [1-2]. Reports of treatment for accidental LN2 injuries are very limited within the current medical literature. To our knowledge, there are three cases, listed within two case reports, describing the management of LN2 burns. These three cases were treated with conservative measures such as dressing changes, demarcation, debridement, amputation, and antibiotics [3-4]. N-acetylcysteine (NAC) is a compound generally used as a hepatoprotective agent, mucolytic agent, or protective anti-inflammatory and antioxidant agent in cases of burns or other tissue damage to prevent further cellular damage [5-6]. There are no available case reports of LN2 injuries treated with NAC. Therefore, we present a unique case in which NAC was used to treat an injury resulting from accidental submersion of the hands in LN2.

## Case presentation

**Figure 1:**
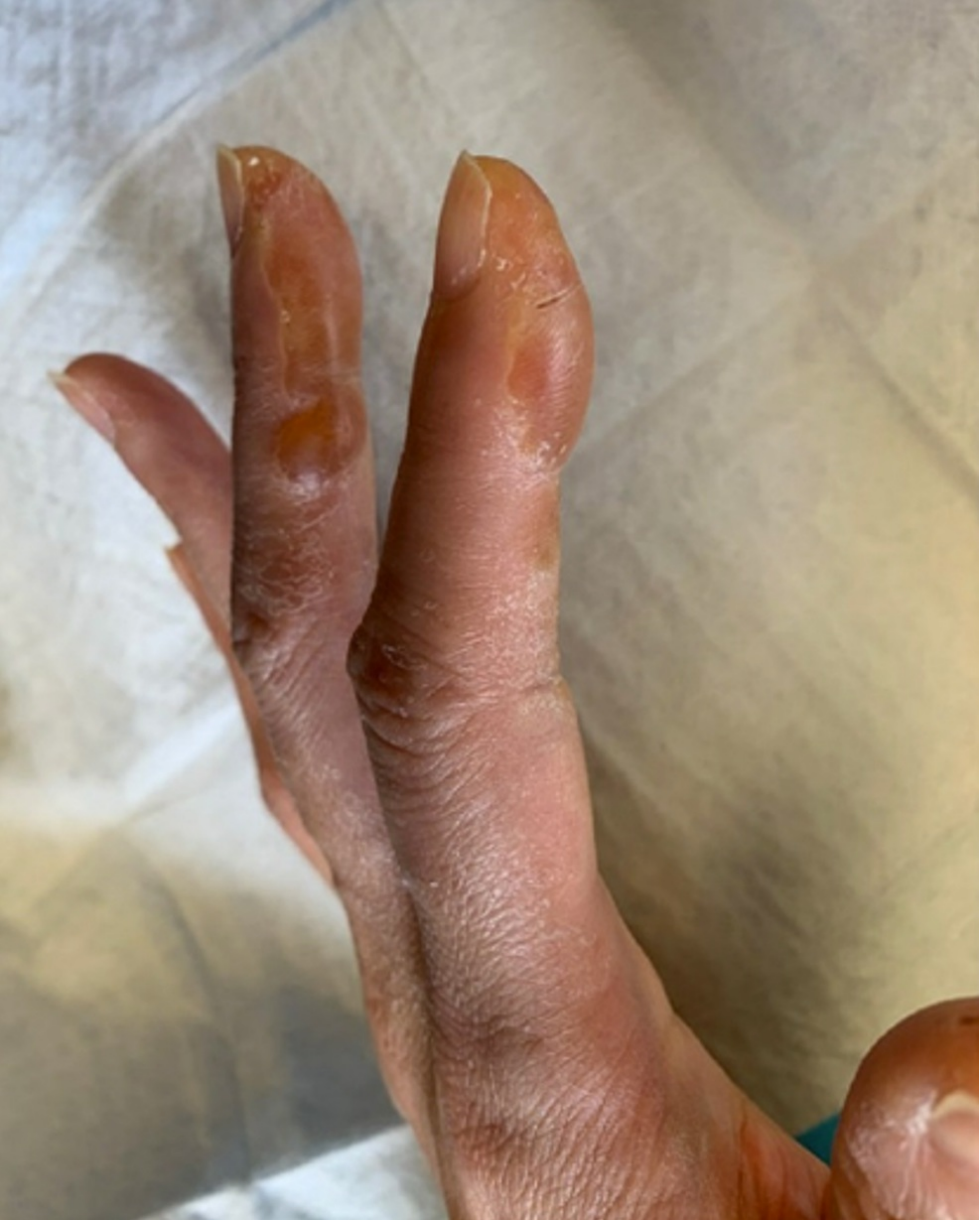
Lateral aspect of the left fingers four days post-injury.

**Figure 2:**
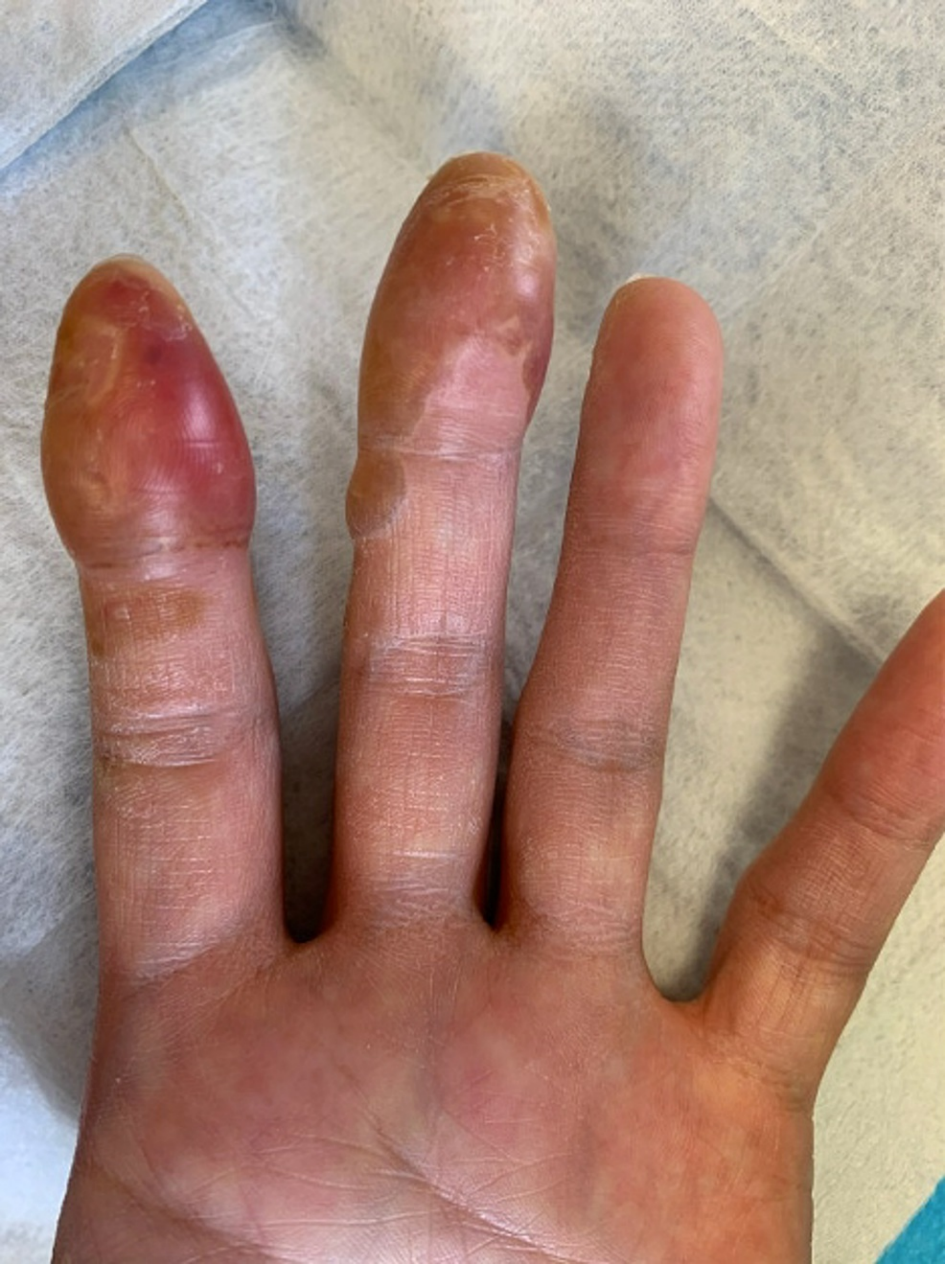
Volar aspect of the left palm and fingers four days post-injury.

A 40-year-old woman with a past medical history of familial hypercholesterolemia presented to the emergency room complaining of pain localized to the left second middle and distal phalanges, left third distal phalanx, and right third distal phalanx following a thermal burn from exposure to LN2. The patient works in research and accidentally dropped a pair of rubber gloves into a container with LN2. She instinctively reached into the container to retrieve her gloves and her fingers contacted the LN2 for approximately 30 s. She immediately noticed pain of 8/10 severity in her left index and middle fingers as well as her right middle fingertip. Upon examination, her left index and middle fingers (Figures 1-2) and her right middle fingertip showed clear blisters. The regions were well perfused. She denied any other symptoms. Bilateral X-rays of the hands and X-rays of the affected fingers showed no acute bony abnormalities. The emergency room team contacted poison control who recommended infusion with NAC. The emergency room began a 16-h IV infusion of NAC 200 mg/mL (20%) 4500 mg in 5% dextrose solution and consulted both orthopedic surgery and vascular surgery, who did not recommend surgical intervention. They chose to admit the patient overnight for observation.

The patient was discharged the next morning after receiving one dose of NAC. At this time, her fingers were swollen and erythematous. Orthopedic surgery recommended a 10-day course of 100 mg oral doxycycline two times per day and expedient follow-up in clinic.

At follow-up four days following discharge, she continued to report pain and throbbing in the affected fingers. On examination, her fingers remained swollen and blistered. Her motor and sensory exam was normal with the exception of loss of sensation to the left index finger distal phalanx in the area of her blister. Capillary refill remained brisk for less than three seconds. The left index finger blisters were lanced with a 15-blade scalpel in clinic, and the patient was given instructions to apply mupirocin ointment two times daily and to follow-up in clinic in one week for another skin check. She continued to return every one to two weeks for regular skin checks. Subsequent exams showed decreased discoloration and swelling as well as improved sensation and range of motion.

**Figure 3:**
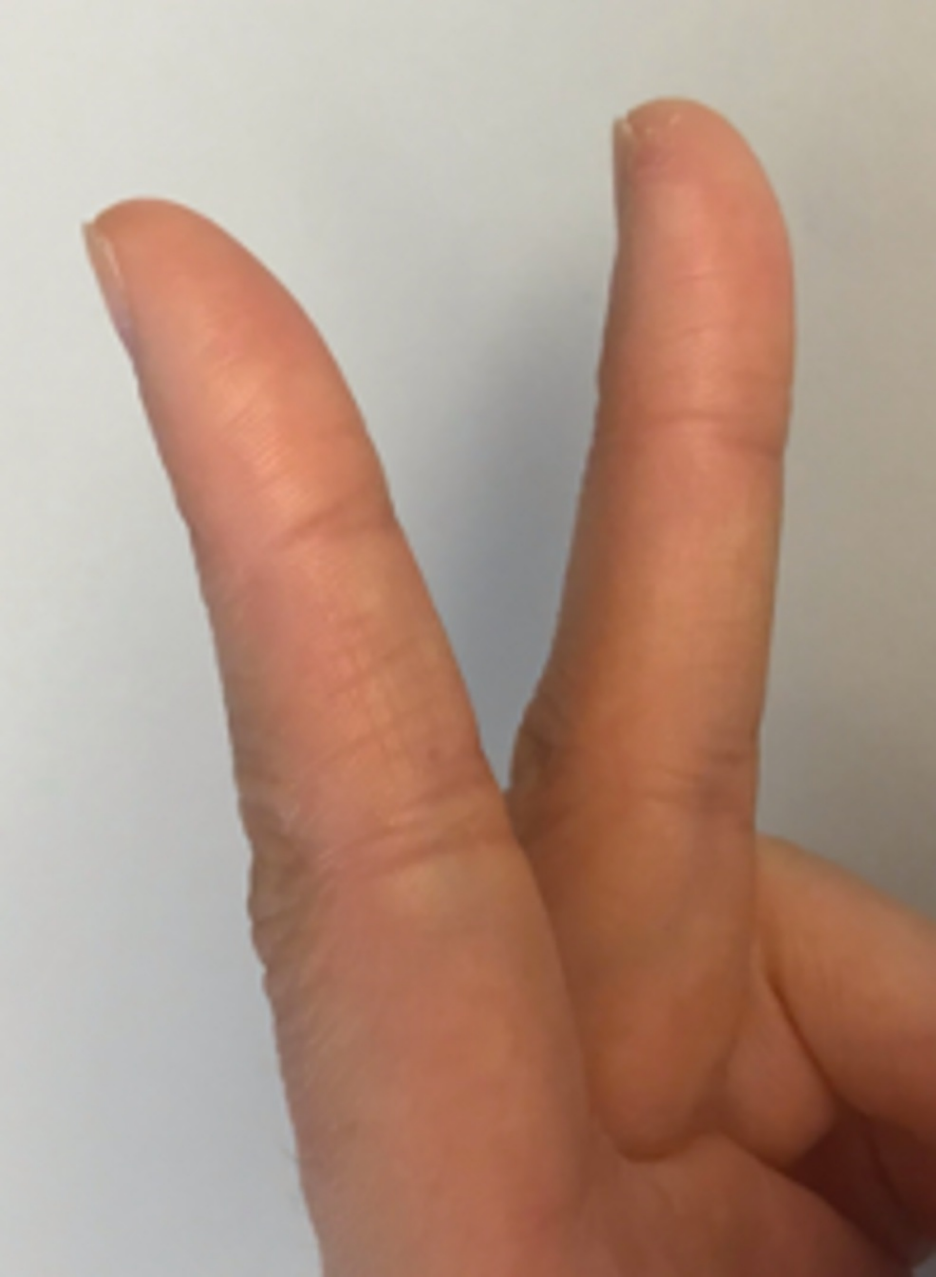
Lateral aspect of the left fingers six months post-injury.

**Figure 4:**
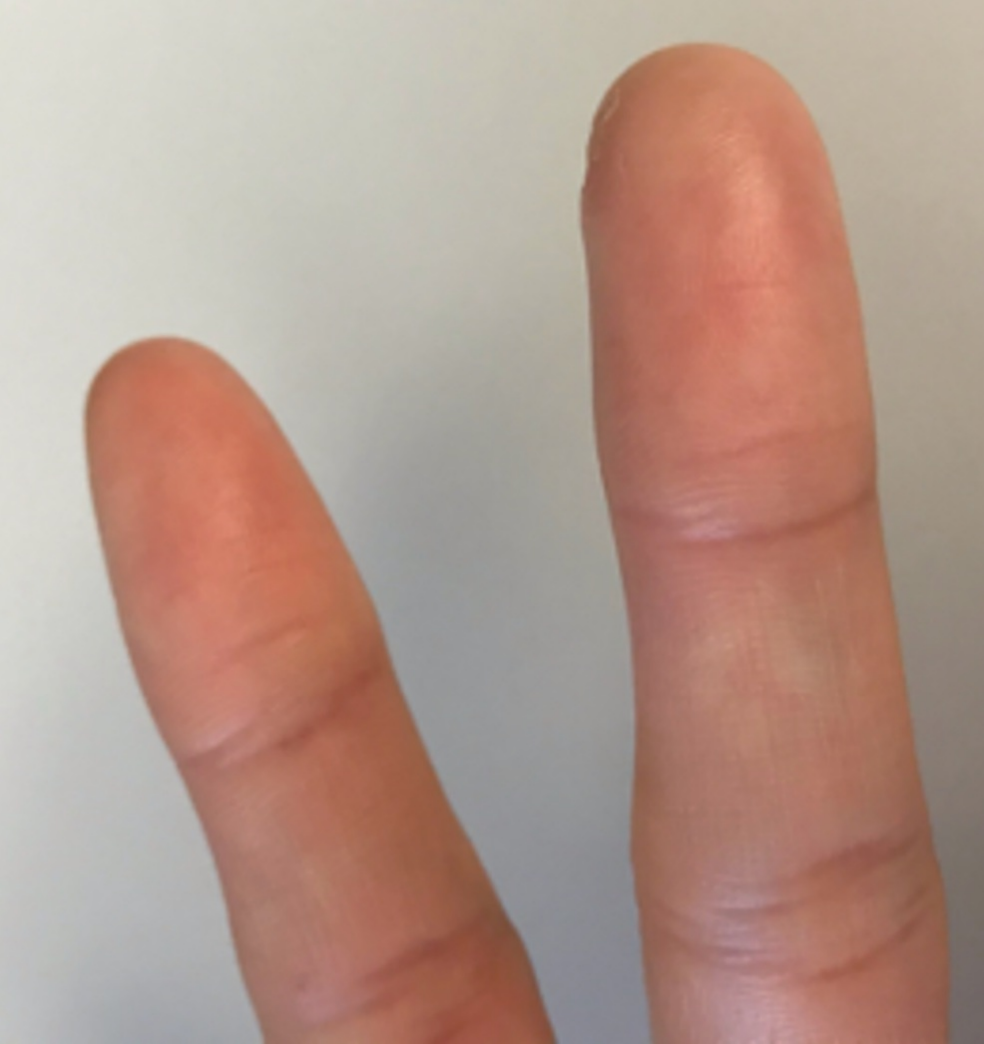
Volar aspect of the left fingers six months post-injury.

The patient was contacted at six months post-injury for final follow-up. She stated that she felt great and had completely normal range of motion. She also stated she had no functional limitations and was able to complete activities such as typing or playing the piano without any trouble. Bilateral DASH scores were obtained, resulting in a score of 0/100 for the right hand and a score of 3.3/100 for the left hand. Images of the patient’s left hand at six months of follow-up are shown in Figures 3-4.

## Discussion

There is limited information to guide the management of accidental LN2 injuries and no established treatment algorithm outside of anecdotal evidence. Previous treatment methodologies have included conservative treatment with dressings, both with and without antibiotics, conservative treatment followed by skin graft, and conservative treatment followed by terminalization of the affected finger [3-4].

Liquid nitrogen is often used for minor dermatologic procedures due to its ability to induce cell destruction, allowing it to effectively combat malignant skin cells [6]. LN2 achieves these effects through two primary mechanisms. It damages vessels within its area of contact, leading to tissue necrosis in the area of blood supply. A subsequent inflammatory effect involving thromboxane A2, prostaglandin F2-alpha, bradykinins, and histamine further contributes to necrosis [7]. Additionally, the rapid cooling of the tissue creates ice crystals between cells, inducing an osmotic effect which draws water into the extracellular space. Crystal formation within cells can also contribute to cell damage. As the crystals thaw, however, the water rapidly returns into the cell, creating swelling within the cell and potentially causing it to burst [6-7]. Though this mechanism proves useful in initiating the destruction of already damaged cells, widespread application can lead to detrimental effects.

This patient was administered NAC as recommended by poison control. NAC has been used successfully in the treatment of acetaminophen overdose, smoke inhalation injury, and burns, among other conditions [5, 8-9]. NAC exerts its healing effects by acting as an antioxidant as well as an inflammatory mediator, making it a reasonable recommendation for conditions where salvage of damaged or at-risk tissues is necessary [4]. At a biochemical level, NAC stimulates adipose derived stem cells and decreases their IL-8 production. Adipose derived stem cells have the potential to assist with wound healing through production of growth factors, however, proinflammatory cytokines such as IL-8 work against them and can worsen wounds [10]. The inhibition of IL-8 combined with the stimulation of these growth-factor-secreting cells further contributes to NAC’s ability to enhance wound healing. This patient was successfully treated with a combination of NAC and wound care, as demonstrated by their full recovery of function, lack of necessity of skin graft, and lack of necessity of amputation. Further research is needed to determine guidelines for use of NAC as treatment for similar injuries in the future.

## Conclusions

Liquid nitrogen is a compound with potential to cause extensive cell injury with prolonged skin contact. NAC can be used to improve outcomes of these injuries by acting as an antioxidant and inflammatory mediator. NAC administration in combination with dressing changes and antibiotics can successfully treat LN2 burns.
